# A Review of Educational Supervision in UK Postgraduate Medical Training: Roles, Responsibilities, and Impact on Trainee Development

**DOI:** 10.7759/cureus.73615

**Published:** 2024-11-13

**Authors:** Omar M Ismail, Umar N Said, Omar M El-Omar, Aryana Jizan, Mohammed A Bhutta

**Affiliations:** 1 Trauma and Orthopaedics, Northern Care Alliance NHS Foundation Trust, Manchester, GBR; 2 Trauma and Orthopaedics, University Hospitals Coventry and Warwickshire NHS Trust, Coventry, GBR; 3 Trauma and Orthopaedics, Calderdale and Huddersfield NHS Foundation Trust, Huddersfield, GBR

**Keywords:** educational supervision, feedback models in medicine, medical education, postgraduate medical education (pgme), postgraduate training, supervisor-trainee relationship, trainee professional development

## Abstract

This review examines the roles and responsibilities of educational and clinical supervisors in the UK postgraduate medical education system, focusing on their impact on trainee doctors’ educational experiences and professional development. Guidance from the General Medical Council and the Department of Health underscores the importance of consistent supervision in various clinical settings. Educational supervisors are critical in helping trainees establish clear, competency-based goals, engage in career planning, and develop resilience. Supervisors are expected to build productive relationships, provide structured feedback, and support workplace-based assessments. Various feedback models, including Pendleton’s and one-minute preceptor models, are discussed for their effectiveness in promoting reflective practice and competence. By fostering open communication and structured assessment, educational supervisors help trainees navigate the complexities of clinical practice and advance in their medical careers. This review outlines the essential supervisory skills, training recommendations, and best practices contributing to effective supervision and trainee success within the UK medical education framework.

## Introduction and background

Educational supervisors are crucial in guiding medical students and trainee doctors during clinical placements. Medical students and trainee doctors must rotate frequently amongst a wide variety of specialties and in different hospitals and community settings. In unfamiliar environments, it is important to have a consistent supervisor who can help guide the mentee. Each stage of training and placement will have specific goals that are relevant to that stage. The Department of Health and General Medical Council (GMC) has long stated the importance of effective supervision [1,2]. However, there can be a disparity in the quality of supervision, particularly as formal training is not given [3]. Supervision can be defined as the provision of guidance and feedback on matters of personal, professional, and educational development in the context of a trainee’s experience of providing safe and appropriate patient care [4]. This demonstrates effective supervision is based on the trainee’s experiences in that unit, and this review will further outline the roles and responsibilities of the effective supervisor (for both educational and clinical supervisors).

In order to retrieve the literature on this topic, using the PubMed platform, keywords such as “supervision”, “medical training”, and “feedback” filtered results. The database selected was MEDLINE. Only literature published after 1980 were included, and was ranked using the Centre for Reviews and Dissemination standards.

Differentiating clinical and educational supervisors

It is also important to discern, when discussing the United Kingdom's postgraduate medical education system that clinical supervisors and educational supervisors play differing roles in trainees' education. The clinical supervisor is usually local to the department and is selected to oversee the trainee’s clinical work in the placement, and changes with each rotation [5]. Conversely, the educational supervisor usually stays with the trainee for the duration of the training program. Their role is to oversee the trainee’s educational progress to ensure they are meeting competencies across the placements [5].

## Review

Training and preparation for supervisors

Before the placement, educational supervisors should have training provided by the medical school, hospital trust, deanery, or royal college as appropriate. Modernising Medical Careers (MMC) was implemented in 2005, and there are clear roles and responsibilities detailed for both clinical and educational supervisors [6]. MMC has outlined the requirement for an educational supervisor to support trainees as they progress through their careers. Therefore, Health Education England (HEE) has recommended various training modules in the ‘Supporting Educators and Learners’ book [7]. This will help to ensure supervision meets a minimum standard for all trainees.

Initial meeting and goal-setting

At the start of the placement, the supervisor and trainee should have an ‘initial meeting’. To help facilitate this, portfolio platforms such as Horus and the Intercollegiate Surgical Curriculum Portfolio (ISCP) have templates of mandatory questions to fill out. For instance, the ISCP form is consistent for all surgical trainees in all grades. It asks the trainee and supervisor to fill out the student’s objectives for the placement in general, and then specifically with regards to outpatient clinics, the unselected emergency take, ward rounds and inpatient care, operating lists, and multi-disciplinary working. The form then asks the supervisor to outline what support the trainee can expect for each objective and how these objectives can be achieved. This can be used to form a ‘Personal Development Plan’, which should have ‘SMART’ (Specific, Measurable, Achievable, Relevant, Time-bound) goals to ensure they are achievable [8].

Providing uniformity is helpful, as the deaneries can ensure that the trainees meet the same goals. The form template can also help the supervisor ensure the trainee makes a clear and concise plan for the upcoming placement. The supervisor can then recap what specific advice and support he can give for each trainee’s goals and ensure the goals are achieved. The supervisor can also discuss the curriculum with the trainee to ensure the trainee is aware of which competencies are required and how many workplace-based assessments are needed.

Career planning and mentorship

In addition to planning out the learning objectives for the placement, it is important to be able to plan out learning objectives for the upcoming months and years. Career planning is an integral part of educational supervision. Medical students and very junior doctors, in particular, may not have much of an idea of future careers or how to go about succeeding in these pathways. For more senior trainees, there is still the question of subspecialisation and gaining a consultant post. The supervisor can help give advice and expertise on preparing applications, maximizing points, and improving their curriculum vitae [9].

Building effective relationships

Finally, in this initial meeting and throughout the placement, it is important to note that there is a human factor in the education supervisor-trainee relationship. It is not acceptable for the supervisor to be seen as a lofty superior whose existence is to assess and discipline the trainee. As with ordinary relationships outside the workplace, there is no ‘one-size-fits-all’ formula for supervisor-trainee relationships [10]. The supervisor should spend time getting to know the trainee to have a relatively relaxed relationship. The supervisor should be seen as a role model for the trainee. A cordial relationship between the trainee and supervisor will make the trainee more receptive to feedback and will be more forthcoming with issues or queries [11].

Furthermore, in the placement, the trainee may struggle and underperform. One of the key roles of the supervisor is to recognize when a trainee is having difficulty, to work with the trainee to find the reasons underpinning this, and to attempt to come to a solution. The trainee may be suffering from difficulties in the workplace (for example, difficulties in grasping the clinical content or personal issues with the workforce), or difficulties could occur on a personal level (such as health reasons or family issues). The educational supervisor should have strong interpersonal skills, empathy, and listening skills and be able to offer pastoral support to the trainee or deal with workplace issues [9, 12]. Supervisors can advise the trainee on how to avoid burnout, develop resilience, and maintain a healthy work-life balance [13].

Providing feedback: Models and approaches

Strong interpersonal skills, teaching skills, and clinical competence are vital assets to deliver effective feedback [12]. When observing the trainee in the clinical environment, the supervisor should provide feedback to help them improve. A widespread method of feedback delivery in Medicine is Pendleton’s model [14]. After the observed clinical activity, the trainee is asked what they felt went well, followed by the supervisor’s thoughts. The trainee is then asked how they could improve next time, followed by the supervisor’s thoughts again. An action plan is then created, and further praise is given. This process is further described as a ‘compliment’ or ‘feedback’ sandwich, where the trainee is reminded of their successes at the end of their conversation to prevent dwelling on the areas of improvement. This will help improve the morale of the individual [15]. When feedback is given, it should be goal-referenced, tangible, actionable, comprehensible, timely, continuous, and consistent in quality, as suggested by [16].

Another crucial method of delivering feedback for the supervisor is the ‘one-minute preceptor’ first described as the “five-step ‘micro-skills’ model of clinical teaching” [17]. The five micro-skills are: 1. Get a commitment - the trainee should articulate a diagnosis; 2. Probe for supporting evidence - discern the trainee’s reasoning; 3. Teach general rules - impart key messages for the topic in question; 4. Reinforce what was done well - feedback on positive aspects; 5. Correct mistakes - provide constructive criticism

The idea behind this is to facilitate quick and effective clinical supervision with the trainee. It has been shown that the one-minute preceptor method is rated highly by trainees and supervisors and improves instructor performance in all the skills listed above when delivering feedback [18].

Workplace-based assessments (WBPAs) and progress monitoring

Feedback is a key component of workplace-based assessments (WPBAs). The General Medical Council (GMC) strongly advocates for WPBAs, and they are a common feature of portfolio requirements for trainees in their yearly appraisal meetings (Annual Review of Competency Progression (ARCP)) [19]. WBPAs are formative assessments that assess performance on skills such as interacting with patients and performing procedural skills. These are used to ensure the trainee is on track to meeting core competencies by the end of the rotation or training program.

Although the onus is on the trainee to get these signed off, there is no reason why the supervisor cannot identify areas where WBPAs can be done. A supervisor with greater knowledge of the department and specialty is more adept at assessing which scenarios benefit WBPAs and can offer the trainee to undertake one. WBPAs typically have a standardized proforma to be filled out, which will further aid the supervisor in providing useful feedback to the trainee. They ensure uniformity for both parties in knowing what competencies are required and to what level of performance for each grade of doctor. At the end of each WBPA is the opportunity to create SMART goals (specific, measurable, achievable, relevant, time-bound) [8]. This format ensures that the goals are not vague and are more likely to be adhered to by the trainee.

WBPAs are one way to monitor a trainee’s progress throughout the placement. The supervisor may have a variable amount of clinical experience with the trainee. A trainee in a breast surgery placement with five consultants and three registrars can expect to work more closely with each consultant compared to a trainee in a trauma and orthopedic surgery department at a major trauma center with 20 consultants. Therefore, at the very minimum, it is important to have a mid-placement meeting to provide an opportunity for concerns to be raised before the end of the placement. Practice is variable in the length and frequency of supervisor-trainee meetings [12]. In general practice meetings, it is common practice to have a weekly supervision meeting for both teaching purposes and to debrief on difficult cases from the last week and discuss concerns. This is a good template for supervisors to aspire towards in other specialties.

End of placement review

At the end of the placement, an end-of-rotation meeting should be held. Progress should be reviewed to date in relation to the curriculum requirements and the learning agreement/personal development plan created at the start of the placement. All required assessments and competencies should be met. If there are any outstanding, then a plan should be made to be able to achieve this in the next placement which may require a new personal development plan. All documentation for the placement should be checked so the trainee is ready for ARCP [20]. The educational supervisor should be aware of the ARCP panel function and of the significance of their report as evidence to that panel. A detailed report is more likely to help the panel come to the correct decision regarding the progression of the trainee and should thus be able to bring all the placement components together into a concise report [20].

## Conclusions

In conclusion, the bulk of the supervisor’s work takes place before and at the start of the placement. Training and preparation are key to ensuring the supervisor is aware of their task and what support they must provide the trainee. At the start of the placement, it is important to establish a strong, trusting relationship with the trainee and to ensure that both parties know what the other expects of them by the end of the placement. Key aspects of effective supervision can be summarised by focusing on long-term career planning, providing feedback on educational and clinical progress, and having frequent meetings to monitor the trainee’s progress. Supervision can be seen as an onerous task by some, but it should be a rewarding task that will develop the next generation of doctors.
